# Role of the adaptive immune response in sepsis

**DOI:** 10.1186/s40635-020-00309-z

**Published:** 2020-12-18

**Authors:** Jack Brady, Shahd Horie, John G. Laffey

**Affiliations:** 1 Anaesthesia, School of Medicine, Clinical Sciences Institute, National University of Ireland, Galway, Ireland; 2 Regenerative Medicine Institute (REMEDI) at CÚRAM Centre for Research in Medical Devices, Biomedical Sciences Building, National University of Ireland Galway, Galway, Ireland; 3 Department of Anaesthesia, Galway University Hospitals, SAOLTA University Health Group, Galway, Ireland

**Keywords:** Sepsis, Immune suppression, Immune homeostasis

## Abstract

Sepsis is a syndrome of shock and dysfunction of multiple vital organs that is caused by an uncontrolled immune response to infection and has a high mortality rate. There are no therapies for sepsis, and it has become a global cause for concern. Advances in patient care and management now mean that most patients survive the initial hyper-inflammatory phase of sepsis but progress to a later immunosuppressed phase, where 30% of patients die due to secondary infection. Deficits in the adaptive immune response may play a major role in sepsis patient mortality. The adaptive immune response involves a number of cell types including T cells, B cells and dendritic cells, all with immunoregulatory roles aimed at limiting damage and returning immune homeostasis after infection or insult. However, in sepsis, adaptive immune cells experience cell death or exhaustion, meaning that they have defective effector and memory responses ultimately resulting in an ineffective or suppressed immune defence. CD4+ T cells seem to be the most susceptible to cell death during sepsis and have ensuing defective secretory profiles and functions. Regulatory T cells seem to evade apoptosis and contribute to the immune suppression observed with sepsis. Preclinical studies have identified a number of new targets for therapy in sepsis including anti-apoptotic agents and monoclonal antibodies aimed at reducing cell death, exhaustion and maintaining/restoring adaptive immune cell functions. While early phase clinical trials have demonstrated safety and encouraging signals for biologic effect, larger scale clinical trial testing is required to determine whether these strategies will prove effective in improving outcomes from sepsis.

## Background

Sepsis is a clinical syndrome defined as ‘life-threatening organ dysfunction caused by a dysregulated host immune response to infection’ [1]. Sepsis is a major health concern and a leading contributor to mortality and critical illness globally [2, 3]. In 2011, it was responsible for more than $20 billion in expenses in all US hospitals [4]. In 2017, global sepsis incidence was estimated at 48.9 million cases and sepsis-associated deaths were estimated at 11.0 million cases [3]. The highest incidence has been observed in young children and the elderly, with the main causes being lower respiratory tract and abdominal infections [3]. The incidence of sepsis is also expected to rise as a result of an ageing population that has numerous comorbidities and an increasingly impaired immune system [3, 5, 6]. Multi-drug–resistant bacterial pathogens are another major challenge meaning that sepsis is becoming increasingly difficult to manage clinically. Patients who survive sepsis are often seen to possess long-term complications including physical, psychological and cognitive impairments with additional negative healthcare and social implications [7].

Septic patients frequently present with fevers, cardiovascular shock and respiratory and/or systemic organ failure [8]. The prototypical clinical features of early sepsis result primarily (but not always) from an overwhelming, ‘pro-inflammatory’ immune response which has led to many drug trials attempting to block these pro-inflammatory effects [9]. However, strategies attempting to dampen this overactive immune response to infection, by blocking interleukin (IL)-1β and tumour necrosis factor (TNF)-α for example, ultimately failed in producing any survival benefit [10]. Over 100 clinical trials with different pharmacological agents have taken place, yet no single FDA-approved therapeutic agent exists that is capable of improving survival in patients with sepsis [11]. Clinical outcomes of patients from the initial hyper-inflammatory phase have improved over the last decade with aggressive source control, earlier appropriate antibiotic therapy, titrated fluid and pressor therapy, and better organ supportive measures, particularly ventilator management [12].

**Fig. 1:**
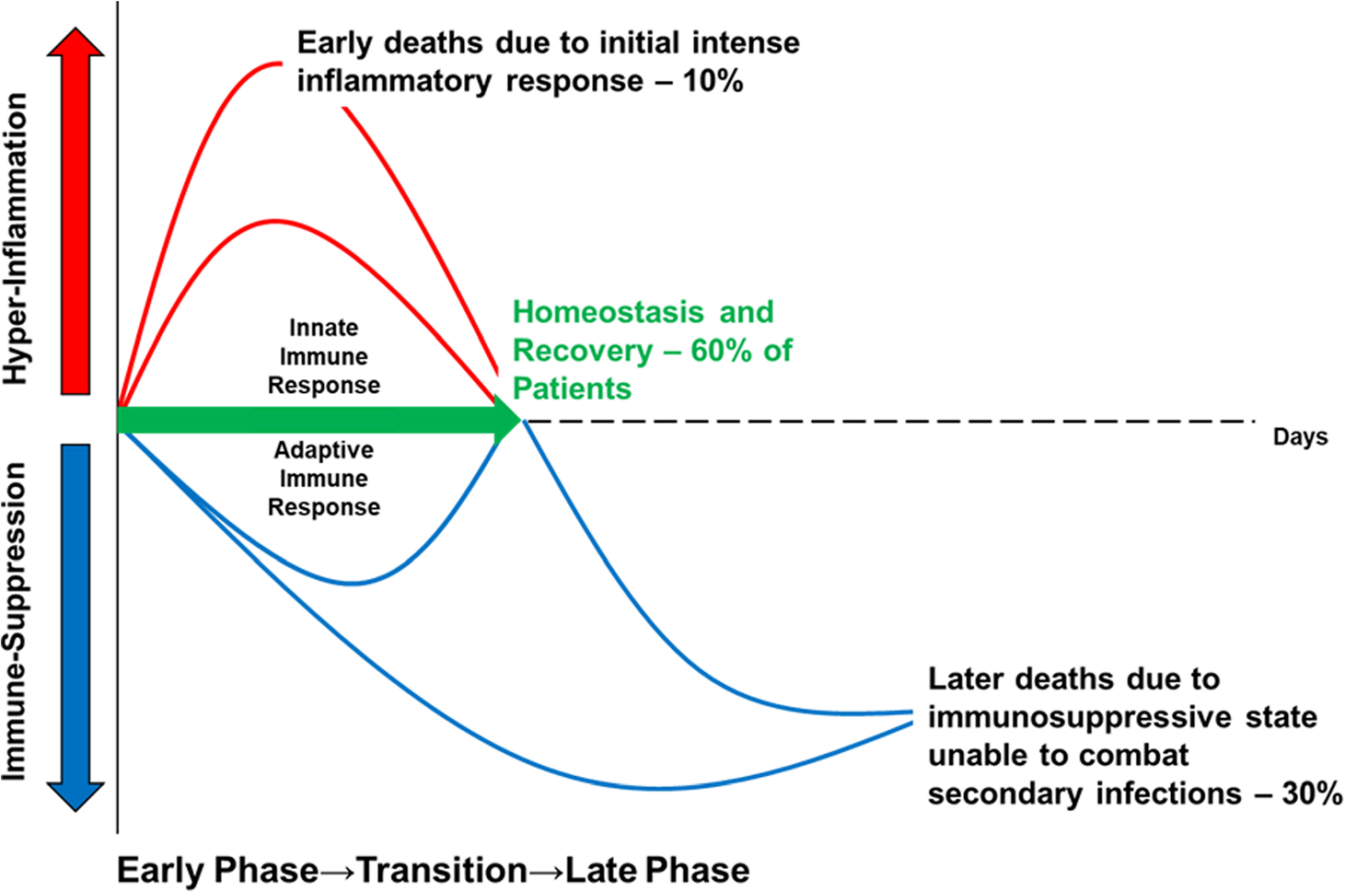
The time course of immune events in Sepsis. Adapted and reprinted from Macmillan Publishers Ltd: Hotchkiss et al. [15]. Nature Review Immunology, Copyright 2013

Unfortunately, despite these improvements, and despite the fact that more people are now surviving the initial stages of septic shock and organ dysfunction, sepsis remains responsible for the most deaths in intensive care units (ICU’s) [12] and long-term mortality is still in the region of 40–80% [13]. This is because patients are now more frequently transitioning into a later state of prolonged immune suppression [14] (Fig. 1).

Impairments in the adaptive immune response increase the likelihood of developing persistent, recurring, secondary and nosocomial infections which often lead to death in sepsis patients [16]. With increasing numbers of patients developing these immune complications, more recent research efforts are now focussing on attempting to understand the underlying changes that occur to both innate and adaptive immune cell populations and highlighting potential new targets for therapy for this syndrome. In this review, we will focus solely on the effects that sepsis has on the adaptive immune system. We will then focus on how some of the immune cells involved in adaptive immunity may be directly targeted to provide potential therapeutic benefit in treating this syndrome and improving long-term survival.

## The innate versus the adaptive immune response

**Fig. 2:**
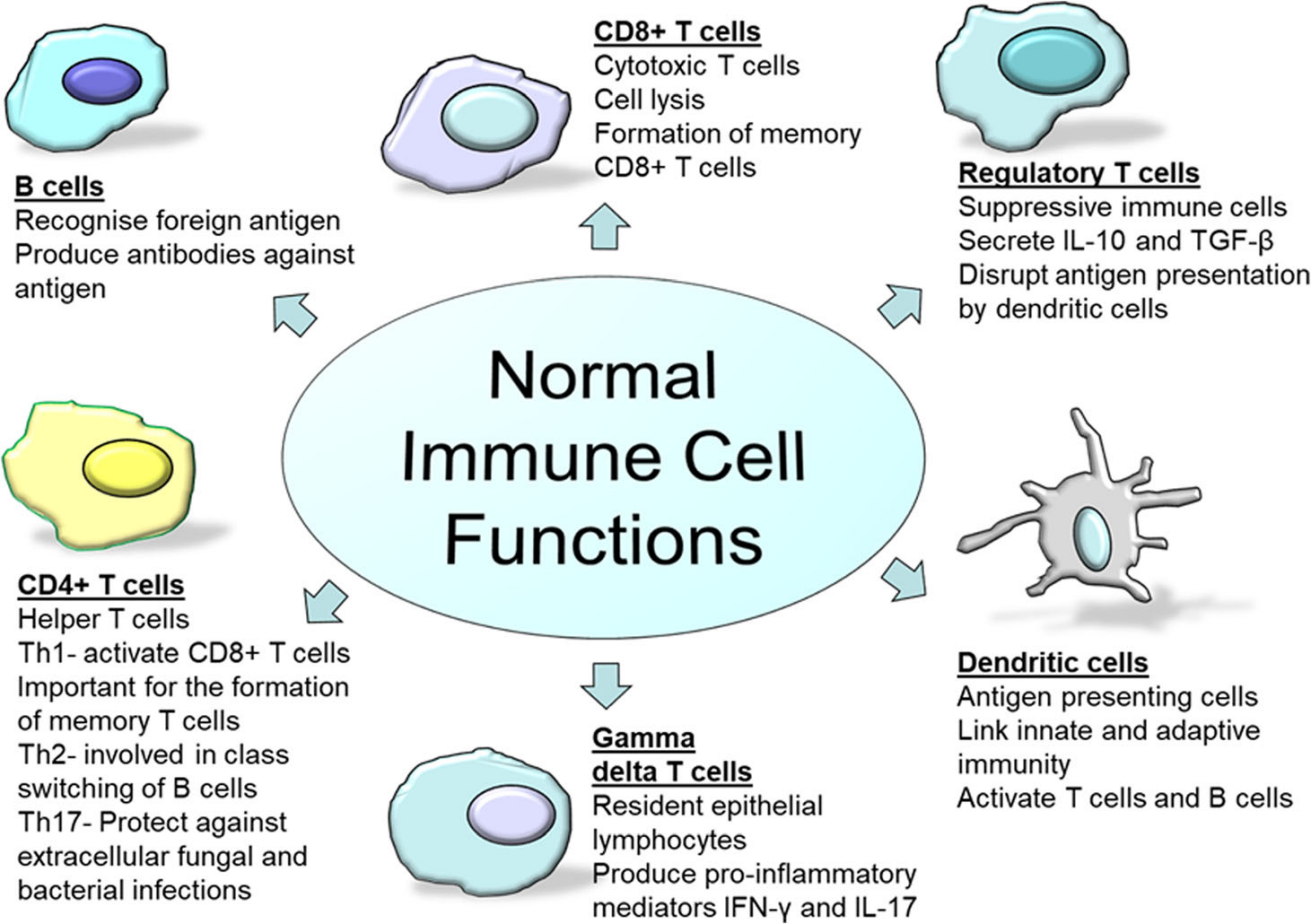
The normal functional state of adaptive immune cells

There are two components of the human immune system, and these are the innate and adaptive immune systems. The innate immune system is the first line of defence against invading pathogens and acts rapidly and non-specifically to fight infection. The adaptive immune system, in contrast, is much slower to respond, but is capable of recognising unique antigens and utilising immunological memory to enhance the immune response following subsequent exposures of the same antigens. Both components of the immune system comprise of a variety of cell types. In the innate system, there are natural killer (NK) cells, mast cells, eosinophils, basophils and phagocytic cells which include macrophages, dendritic cells (DCs) and neutrophils. The adaptive immune system relies on fewer cell types to carry out its operations, and these are lymphocytes, namely T cells and B cells. B cells are known to be important producers of antibodies and plasma cells necessary for long-term immune protection, while T cells can be classified further into a number of different subclasses, each with unique functions, and these include CD4+, CD8+, gamma delta (γδ) and regulatory T cells (Tregs) (Fig. 2).

## Adaptive immune cell functions

CD4+ T cell activation results in rapid polarisation into CD4+ subsets which are T helper (Th) 1, Th2 or Th17. The Th1 class is important for the activation of CD8+ T cells and for the formation of memory T cells via IL-2 secretion [17, 18]. Th2 cells are involved in class switching of B lymphocytes via IL-4 and IL-5 secretions [19], and Th17 cells are effector cells that produce IL-17, IL-22 and TNF-α primarily in response to extracellular fungal and bacterial pathogens [20]. In addition, CD8+ T cells are responsible for the clearing of an infection and for the generation of memory CD8+ T cells in response to infection or vaccination [21]. Once CD8+ T cells bind their cognate antigen in the presence of co-stimulatory molecules and cytokines, they undergo rapid proliferation and expand in number rapidly to gain effector functions [21]. Effector functions include cytokine secretions (including interferon (IFN)-γ and TNF-α) and the ability to lyse cells [21].

γδ T cells are the prototype of ‘unconventional’ T cells. They make up a relatively minute subset of T cells in the peripheral blood. Unlike the more common CD4+ helper and CD8+ cytotoxic T cell populations containing a T cell receptor composed of classic α and β chains, these cells possess a receptor composed of γ and δ chains. They are the most abundant epithelial lymphocytes in the lung (8–20%) and intestine where they promote immune homeostasis [22]. In the lung, they are known to protect against pneumonia infections [22], and they are considered the initial line of defence in the mucosa of the intestine [23]. Once activated, γδ T cells release pro-inflammatory mediators including IFN-γ and IL-17 which aid against infection.

Tregs are another important subset of T cells that generally represent 5–10% of total CD4+ T cell populations in the peripheral circulation and lymphoid compartments [24]. These cells play a pivotal role in immune homeostasis by not only resolving inflammation after infection but also suppressing excessive adaptive immune responses [25]. They also work in order to maintain self-tolerance and avoid autoimmune disease [25]. They function through the secretion of suppressive cytokines including IL-10 and TGF-β, but also through cytolysis, metabolic disruption and through targeting antigen presentation by DCs [25].

It has become clear in recent years that the dichotomy between innate and adaptive immunity may indeed be an over-simplification, as some cell types are now known to display functions relating to both arms of immunity. This is particularly true for DCs which play an important role in linking innate and adaptive immunity through the process of antigen presentation [26]. They are responsible for maintaining immunological tolerance by migrating to draining lymph nodes and presenting self-antigens to lymphocytes [27]. The presence of mature DCs is also crucial as they promote the migratory potential, cytokine secretion and activation of T cells and can initiate antigen-specific antibody responses in naïve B cells [28].

## Adaptive immune response dysfunction in sepsis

**Fig. 3:**
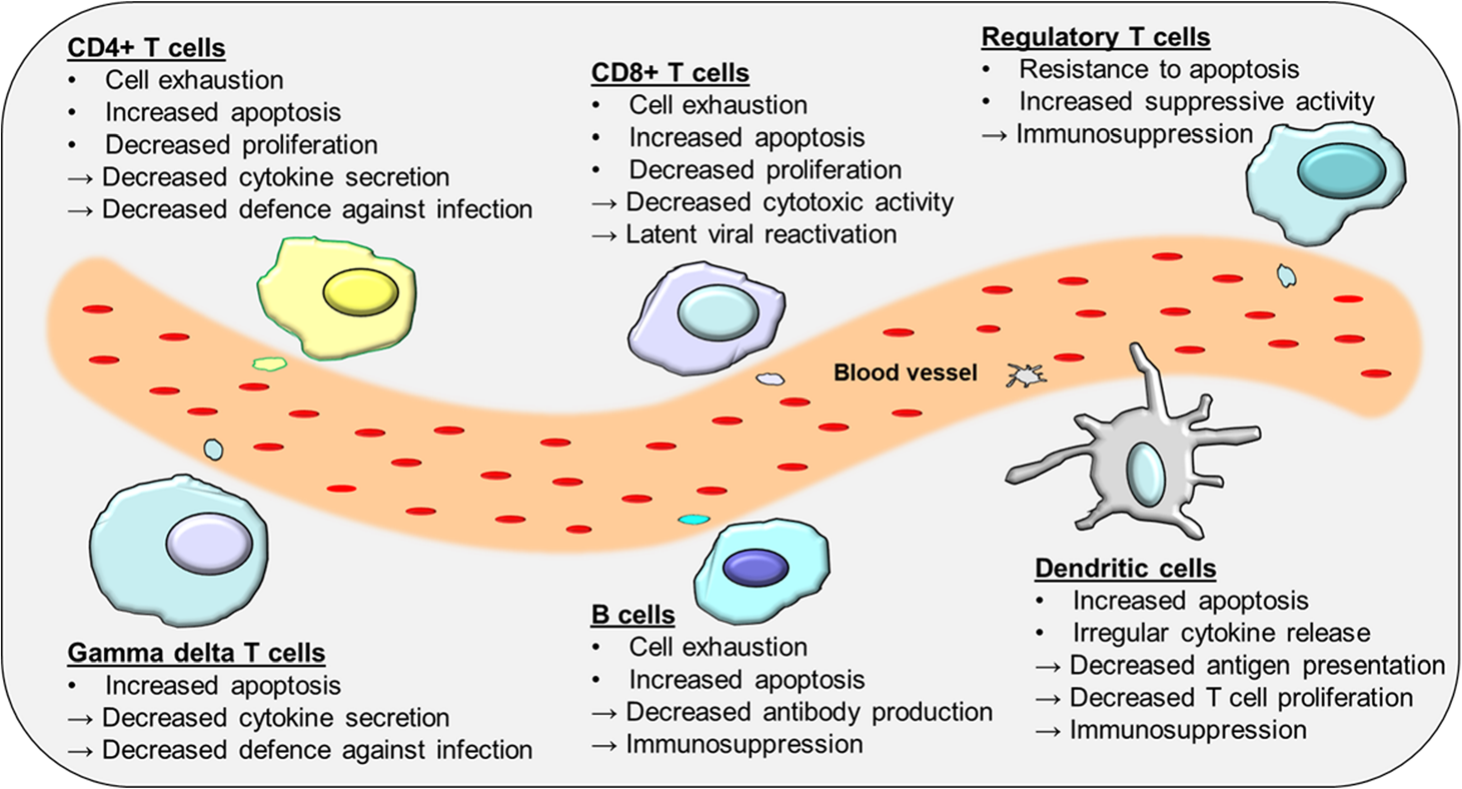
Adaptive immune cell function is dysregulated in sepsis

The adaptive immune response as previously mentioned has a number of roles. It is important for limiting inflammation and tissue damage after an infection and returning overall host immune homeostasis through a number of mechanisms. In sepsis, these processes and functions are disrupted or become dysregulated leading to an improper defence against infection and/or immune suppression; these are discussed below (Fig. 3).

### Cell death

Apoptosis is an important mechanism of regulating immune homeostasis. It can also play a detrimental role in disease pathology. Apoptosis has been shown to be responsible for causing major depletion of immune cells, including CD4+ and CD8+ T cells, B cells and DCs in multiple organs from patients who died from sepsis [29–31]. This process of cell death has been observed to occur in both preclinical models and in human septic patients [32, 33]. This depletion of cells has been shown to occur across all age groups and in response to different classes of microorganisms causing infection [29, 34, 35]. Post-mortem analyses have demonstrated this elevation of apoptosis in the spleens of sepsis patients 90 min post-death compared to non-septic controls [29]. Immunohistochemistry analysis in these samples has also confirmed significantly higher levels of caspase 3 activity which is indicative of the apoptotic process [29].

Other studies have shown that both CD4+ and CD8+ T cells in the peripheral blood are also more prone to undergo apoptosis in septic patients when compared to non-septic controls [36]. T cells when isolated from septic patients have increased levels of caspase 8 and caspase 9, indicating that the death process occurs by both the intrinsic and extrinsic pathway [32]. Furthermore, genes encoding pro-apoptotic proteins Bim, Bid and Bak have been observed to be overexpressed in the peripheral blood of patients with sepsis [37]. This apoptosis of T cells and increased expression of pro-apoptotic proteins contributes to the chronic state of immune suppression in these patients and provides an explanation for the enhanced susceptibility to secondary infections and latent viral reactivation.

### T cell exhaustion

T cell exhaustion is a state of T cell dysfunction that was first described in mice that suffered from chronic viral infections and has since been indicated with septic patients and patients with other immunocompromised diseases such as HIV and cancer [38]. In sepsis, the sustained antigen load with extremely elevated levels of both pro- and anti-inflammatory cytokines constitutes an ideal environment for the development of T cell exhaustion [39].

One study, in which the spleens of sepsis patients were isolated following death, demonstrated substantial evidence to support the concept of sepsis-induced T cell exhaustion [14]. There was an evident loss of inflammatory cytokine IFN-γ and TNF-α production from T cells following stimulation, an increase in the expression of PD1 on CD4+ T cells and a drop in the levels of CD127 on T cells which is characteristic of exhausted T cells [14]. Macrophages were also shown to possess elevated levels of programmed cell death ligand 1 (PD-L1) [14]. Other studies identified that an elevated level of PD1 on circulating T cells from individuals with sepsis is associated with a reduction of the proliferative capacity of T cells and an increase in the susceptibility to secondary infections, and this is correlated with mortality [40]. Inhibiting the interaction of PD1 and PDL1 in a variety of clinically relevant animal models of sepsis has been shown to improve survival rates and highlights the crucial involvement of this pathway with poor disease outcome [41–43].

### CD4+ T cells

Overall, it appears that CD4+ T cells are the subset which are most affected in sepsis patients [32, 44, 45] (Fig. 3). CD4+ T cells undergo the most significant amount of programmed cell death, and survivors of the disease demonstrate prolonged reduction in this population of cells [14, 30]. Studies have observed a decrease in the production IL-2, IL-12 and IFN-γ from both Th1 and Th2 populations in sepsis patients [46–48], and these cells persist in displaying significant reductions in their secretory profiles in sepsis survivors [49]. The protective effect of Th17 cells is also diminished following the onset of sepsis [50] as is their cytokine response [51]. This reduced effector capacity of Th17 cells may negatively impact outcome in sepsis by increasing the susceptibility of patients to bacterial and fungal infections [20, 52].

### CD8+ T cells

Studies have highlighted that the reactivation of latent viruses is known to be associated with immunocompromised individuals with sepsis [53, 54]. In a study looking at the activity of T cells in severe sepsis patients with human cytomegalovirus (HCMV), it was observed that there was impaired poly-functionality of CD8+ T cells [54]. Furthermore, the relative frequency of CD8+ T cells was significantly reduced in patients who had HCMV reactivation [54]. These patients also showed enhanced PD1 expression on their T cells compared to patients without the virus reactivation [54], which is another example of T cell exhaustion. The expression of PD1 was inversely proportional to the number of poly-functional CD8+ T cells [54].

### γδ T cells

Depletion of γδ T cells is associated with higher mortality rates in critically ill sepsis patients [23]. As mentioned previously, these cells are required for protection against infections in the lung [22]. The reduction of this cell population within the gut mucosal layer may also in fact result in previously non-threatening bacteria in the mucosa becoming more invasive and entering the blood system to then cause further infections in sepsis patients [55]. Along with reduction in cell number, there has been an observed phenotypic change and impaired function of γδ T cells in patients with sepsis [56]. Liao et al. noted that in comparison with control patients, γδ T cells in septic patients had increased expression of the early activation marker CD69, decreased expression of the recognition receptor NKG2D and increases in both pro-inflammatory and anti-inflammatory mediators [56]. Interestingly, they noted that following antigen stimulation, there was a significant decrease in CD69 and IFN-γ expression [56]. This profile was more evident in non-survivors than in survivors and led the authors to conclude that lower expression of IFN-γ expression upon stimulation is a strong indicator in patient 28-day survival rates [56].

### Tregs

In sepsis, Tregs can potentiate immune suppression by further reducing other effector T cell numbers and functions. Unlike other effector T cells which are more likely to undergo apoptosis [57], Tregs have increased expression of the anti-apoptotic protein Bcl-2 [58] and can evade cell death (Fig. 3). The increase in Treg number and function has been demonstrated in both clinical [59, 60] and experimental studies [61, 62]. This increase has been observed early after the onset of sepsis and is persistent in those who died as a result [63]. Furthermore, there is an increase in heat shock proteins and histones in sepsis which are known to be strong inducers of Tregs [64]. The increased proportion of Tregs in sepsis has a negative effect on normal T cell proliferation and function and contributes to worse outcome [58], most likely because they can prevent the already weakened immune system from mounting an appropriate immune response against secondary infections.

The development and function of Tregs is regulated by FOXP3, considered to be the control gene of these cells. Along with evidence to support increased levels of Tregs in sepsis patients, there is also evidence to support that expression levels of FOXP3 is enhanced in these patients [65]. In a CLP model of sepsis, it was shown that adenosine is largely responsible for the high expression of FOXP3 [66]. TLR4 has also been implicated to affect the activity of Tregs [67]. Cao et al. showed that Tregs from wild-type mice exhibited enhanced secretion of anti-inflammatory cytokines, whereas this effect was attenuated in TLR4^−/−^ mice [67]. The results from this study indicate that TLR4 deficiency can improve immune paralysis by attenuating Treg activity and by restoring a pro-inflammatory cytokine balance. Modulating the activity of TLR4 may be a useful tool in preventing immune suppression in sepsis patients. However, it is important to highlight that although Tregs can contribute to immune suppression in sepsis, Treg depletion does not improve mortality rates. In fact, one study observed the mice deficient in Tregs could not fight the initial sepsis infection [68]. Hence, it is clear that a delicate balance of Tregs is required to maintain immune homeostasis in sepsis.

### B cells

B cells play a pivotal role in combating bacterial infections, but B cell dysfunction is very evident in sepsis [69]. In one study, the percentage of exhausted CD21+ B cells was significantly higher in patients with acute sepsis in comparison to healthy donors [70]. IgM in the serum of patients over the age of 65 was also negatively correlated with health outcomes [70]. Conversely, stimulating B cells from septic patient’s ex vivo resulted in significant reduction of IgM levels in the supernatant [70]. This is an interesting finding as elderly patients with decreased IgM production are likely to be more prone to infection with gram-negative bacteria and fungi [70]. The likelihood of contracting secondary infections associated with sepsis patients has been linked to reduced immunocompetent B cells [70].

Other reports have observed further alterations in B cells derived from patients with septic shock versus age-matched healthy controls [71]. In an ICU study, a low percentage of CD23+, the receptor for IgE, and a higher percentage of CD80+ and CD95+ (indicative of apoptosis) on the surface of B cells was associated with higher levels of mortality in patients with sepsis [71]. A study by Shankar et al. showed that lymphopenia was associated with significantly lower absolute B cell counts and a selective depletion of memory B cells [72]. This depletion of memory B cells contributes to the suppressed immune state experienced by septic patients [72]. Finding methods to reverse this depletion may be beneficial in improving survival outcome for these patients.

### Dendritic cells

Impairment in normal DC function appears to occur in sepsis patients and may be implicated in sepsis-associated immune suppression. The number of DCs is indeed reduced in patients with sepsis; however, the differentiation of monocytes into DCs is accelerated [31, 73], and it has been demonstrated that IL-10–treated DCs are able to suppress the activation of T cells and cause T cell anergy [74]. DCs derived from sepsis patients display an irregular secretory profile that further facilitates the development of immune tolerance [75]. DCs from septic patients have been shown to have a decreased productive capacity of pro-inflammatory cytokines and intracellular cytokine staining following LPS stimulation [76] while IL-10 secretions were shown to be enhanced [76]. Additionally, HLA-DR expression was reduced on all monocyte and DC subsets indicative of immune paralysis, and this observation was long-lasting [76]. Finally, apoptosis of DCs like other cells types has been seen in both human and in animal models of sepsis [77, 78]. Recently, it was shown that overexpression of BCL-2 could prevent depletion of DCs [79], and preventing DC death in mice was shown to offer resistance to endotoxin-induced sepsis [80].

## Long-term immune suppression in sepsis patients

Though it is understood that sepsis survivors are burdened with increased mortality and morbidity several years after developing sepsis, the precise role of sepsis-induced immune suppression in mediating these poor outcomes is not well understood. An animal study investigating the quantitative and qualitative recovery of T cells 3.5 months after sepsis found that despite a rapid recovery of T-lymphocytes, long-lasting impairments in the CD4+ T cells were prominent [81]. Specifically, impairment of Th cell responses against a fungal antigen was noted 1 month after sepsis, and this was thought to be directly due to the reduced number of antigen-specific Th cells [81].

Another 5–10-month follow-up study in sepsis patients showed an increased frequency of circulating FOXP3+ T cells, along with higher concentrations of IL-33 and IL-10 in their serum when compared to healthy controls [82]. It has been observed that mice deficient in the receptor for IL-33 show improved survival post sepsis compared to naïve wild-type mice [82]. IL-33 release is involved in the polarisation of anti-inflammatory M2 macrophages that significantly release IL-10 that in turn aids in the expansion of Tregs and ultimately contributes to the immune suppressed phase of sepsis [82]. Targeting IL-33 could be another potential mechanism to treat sepsis-induced immune suppression.

## Preclinical studies of interventions to reverse immune deficits in sepsis

Multiple laboratory strategies targeting different aspects of adaptive immunity have demonstrated therapeutic potential in overcoming immunosuppression in preclinical sepsis models (Table 1). Strategies including the use of transgenic and knockout mice along with the use of anti-apoptotic agents have demonstrated that by preventing lymphocyte apoptosis, there is an improvement in survival rates [83, 86, 90, 91]. Transgenic mice with T cell overexpression of Bcl-2, a potent anti-apoptotic protein, were protected against sepsis-induced T cell apoptosis in the thymus and spleen and had greater levels of systemic inflammatory cytokines and higher survival [83]. Another study examining selective inhibition of caspase-3 prevented lymphocyte apoptosis and improved overall survival of septic mice [84]. Polycaspase inhibition produced similar results highlighting the importance of caspase-3 activity [84].

**Table 1:** Preclinical evidence that suggests preventing immune cell apoptosis or improving immune cell survival has a positive impact on sepsis mortality rates

Model	Intervention	Effect on immune system	Effect in model
Cecal ligation and puncture (CLP) in C3H-HeJ Mice [83]	Overexpression of Bcl-2 on T-lymphocytes	-Protected from sepsis-induced T-lymphocyte apoptosis in thymus and spleen -Decreased splenic B cell apoptosis	-Improved animal survival -Decreased bacterial burden
CLP in ND4 mice [84]	Selective caspase-3 inhibition or polycaspase inhibition via small molecule inhibitor	-Prevented lymphocyte apoptosis	-Decreased bacteraemia -Improved survival
CLP in C57BL/6 [43]	Anti-PD-L1 antibody	-Prevented sepsis-induced lymphocyte apoptosis -Increased TNF-α and IL-6 production -Decreased IL-10 production	-Enhanced bacterial clearance -Improved survival
CLP in mice [41]	Knockout of PD-1	-Resistance to sepsis-induced cellular dysfunction -Suppressed inflammatory cytokine response	-Decreased bacterial burden -Improved survival
CLP in CD1 mice [42]	Anti-PD-L1 antibody	-Prevented sepsis-induced immune-depletion of lymphocytes and DCs by blocking apoptosis	-Increased Bcl-XL -Improved survival
CLP in CD-1 or C57/BL6 mice [85]	Anti-CTLA4 antibody	-Reduced sepsis-induced apoptosis	-Improved survival at low doses
CLP in C57BL/6 mice [86]	Intra-thymic injections of recombinant adenovirus construct expressing human IL-10	Reduced thymocyte apoptosis	-Increased Bcl-2 expression -Reduced caspase-3 activity -Reduced bacteraemia -Increased survival
CLP or *Pseudomonas aeruginosa* pneumonia in CD-1 mice [87]	Recombinant IL-15	-Blocked sepsis-induced apoptosis of NK cells, DCs and CD8+ T cells -Increased circulating NK cell production of IFN-γ	-Increased anti-apoptotic Bcl-2 -Decreased pro-apoptotic Bim and PUMA -Increased survival
CLP in C57BL6 or CD1 mice [88]	Recombinant human IL-7	-Improved the delayed type hypersensitivity response -Increased absolute splenic counts, proliferation and activation of CD4 and CD8 T cells -Increased expression of the adhesion molecule leukocyte function–associated antigen-1 -Reversed T cell defect in cytokine production	-Improved survival -Decreased tissue fungal colony counts in liver
CLP in C57BL6 or CD1 mice [89]	Recombinant human IL-7	-Blocked apoptosis of CD4 and CD8 T cells -Restored IFN-γ production -Improved effector cell recruitment to infected site -Prevented loss of delayed hypersensitivity -Increased expression of leukocyte adhesion molecules LFA-1 and VLA-4	-Increased Bcl-2 -Improved survival

Another mechanism to prevent lymphocyte apoptosis in preclinical models of sepsis that produced exciting results was the inhibition of PD-L1 [43]. PD-1 is a co-inhibitory receptor that can be expressed primarily on activated CD4+ and CD8+ T cells. Its ligand, PD-L1, is expressed broadly on immune cells. Together, this pathway plays an important role in the regulation of autoimmunity. It has been found that expression of PD-1 is upregulated on T cells, B cells and monocytes following sepsis and that blocking this pathway using an anti-PD-L1 antibody significantly improved survival in CLP mice [43]. Additionally, sepsis-induced lymphocyte depletion was ameliorated, levels of circulating pro-inflammatory cytokines TNF-α and IL-6 were increased, anti-inflammatory IL-10 levels were decreased and bacterial clearance was enhanced [43]. Similar studies investigating PD-1 deficiency [41] or anti-PD-1 antibodies [42] also demonstrated survival benefits in murine models of the syndrome. Another antibody approach involved the blockade of cytotoxic T-lymphocyte antigen-4 (CTLA-4) in a similar concept to blocking the PD-1-PD-L1 pathway [85]. CTLA-4 acts to oppose CD28 which is a critical regulator of early T cell activation and proliferation, and CTLA-4 is found to be upregulated on CD4+, CD8+ and Tregs following sepsis [85]. Here, an anti-CTLA4 antibody showed no effect on inflammatory cytokines but animal survival was increased with low doses [85].

Certain cytokines have also been trialled in these animal models to reverse sepsis-induced immunosuppression and lymphocyte apoptosis. One group reported positive results by selectively targeting thymocyte apoptosis using an adenovirus overexpressing IL-10 [86]. This was found to reduce blood bacteraemia and improve survival due to an increase in Bcl-2 expression and reduction in caspase-3 activity [86]. Of note, systemic administration had no effect on survival. Another study investigating recombinant mouse IL-15 (a pluripotent cytokine that signals cells of both the innate and adaptive immune systems) showed that it inhibited the apoptosis of NK cells, DCs and CD8+ T cells and improved survival in two different models of sepsis [87]. IL-7 has also been investigated due to its potent Bcl-2–inducing anti-apoptotic abilities necessary for lymphocyte survival and expansion. In these studies, it was found that IL-7 treatment protected against lymphocyte apoptosis, restored T cell function and improved survival in animal models of sepsis [88, 89].

## Clinical studies targeting the adaptive immune response

**Fig. 4:**
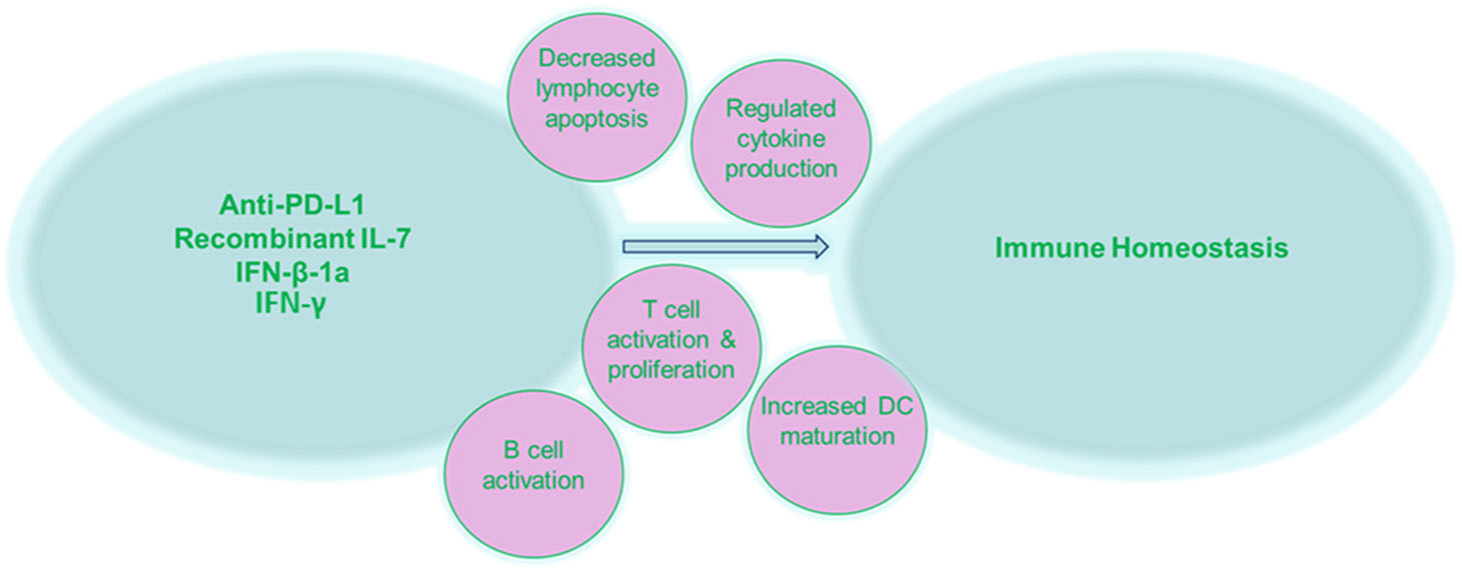
Promising targets for sepsis therapies

Early phase clinical studies using monoclonal antibodies (mAbs), recombinant interleukins and IFN therapies, aimed at targeting adaptive immune responses, have potential for sepsis therapy (Fig. 4). mAbs targeting the PD1-PD-L1 pathway have already been licenced for therapeutic use in cancer and have shown their effectiveness in reducing tumour burden [92]. As mentioned previously, this pathway is also upregulated in sepsis and sepsis does indeed immunologically bear many similarities to cancer [93] and as such this pathway could be targeted to improve outcome. Additionally, blocking this pathway in preclinical sepsis studies [42, 43, 94] as well as ex vivo human sepsis studies [94] has built a solid case for clinical investigation.

A recently completed phase 1b trial looking at immune checkpoint inhibition in patients with sepsis, organ dysfunction and absolute lymphocyte counts (ALCs) ≤ 1100 cells/μl has demonstrated promising results [95]. BMS-936559, an anti-PD-L1 human immunoglobulin G4 mAb which has already been used in patients with HIV [96] and cancer [97], was given to adults with sepsis-associated immunosuppression. The primary objective was to assess safety and tolerability over 90 days following a single-dose administration. BMS-936559 was well tolerated, and there was no observed induction of any ‘cytokine storms’ which is a theoretical risk of any agent that inhibits immune checkpoint pathways [95]. Biological efficacy was also examined, and the investigators found that monocyte deactivation was reduced at the highest doses of treatment, as demonstrated by an increase in monocyte human leukocyte antigen-DR (HLA-DR) expression which was shown to persist past 28 days [95].

The IRIS-7 randomised clinical trial by the same group adopted a different approach to restore immune function [98]. This trial used a recombinant human IL-7 (CYT107) in septic patients in hopes of reversing the profound lymphopenia that is commonly observed [99]. IL-7 treatments have previously been shown to be safe and effective at boosting CD4+ and CD8+ T cell counts in oncologic and lymphopenic patients [100–102]. In this trial, it was shown that IL-7 treatment was well tolerated in septic patients and increased absolute lymphocyte counts (by 3 to 4 fold) and CD4+ and CD8+ T cells, and persistently so, 4 weeks after administration [98]. This data along with the multiple preclinical studies that have demonstrated improved survival following IL-7 administration in septic rats is highly encouraging and calls for next phase clinical testing.

Interferon therapy may be promising for sepsis. In the presence of IFN-β-1a, a type I interferon, DC maturation is enhanced which leads to the activation of T and B cell responses, and at the same time, DCs can limit inflammatory cytokine secretion from CD4+ T cells thus ensuring a state of immune homeostasis [103, 104]. A phase I/II open-label study to examine the effects of IFN-β-1a in acute respiratory distress syndrome (ARDS) patients showed enhanced 28-day survival [105], but unfortunately, the recently completed phase III stage of this IFN-β-1a study showed no clinical benefit [106]. Similar to the early phase of sepsis, ARDS is characterised by hyper-inflammation, and these two syndromes are closely connected in certain clinical scenarios [107]. As such, it would be of interest to investigate whether IFN-β-1a has therapeutic potential instead, for sepsis patients, considering reduced IFNs are implicated in the pathogenesis of the syndrome, particularly in the late immunosuppressed phase.

IFN-γ, which is a type II interferon and cytokine that aids in fighting infection, is lowered in sepsis patients. IFN-γ was recently used as an adjunctive therapy and observed to improve clinical outcome and restore immune responses in a case series of 18 immunosuppressed sepsis patients [108]. The sepsis patients were selected based on the fulfilment of a number of criteria indicative of immune suppression, namely the number of days following the onset of sepsis, monocytic HLA-DR expression, lymphocyte profiles, IL-6 and IL-10 levels, bacterial cultures and clinical scores [108]. Further, larger studies are warranted to verify the therapeutic potential of IFN-γ therapy in immunosuppressed sepsis patients.

## Future directions: challenges and opportunities

Recent research advances have highlighted that sepsis-induced immune suppression contributes to the high mortality rates observed in sepsis patients. Key defects in the adaptive immune response are seen as sepsis progresses, including apoptosis and dysfunction (e.g. reduced cytokine production) of key T cell subsets. This ‘exhaustion’ of T cells also allows invading pathogens to survive and replicate which can lead to an increasing susceptibility of patients to the contraction of secondary infections.

### Ageing and the adaptive immune response

Immunosenescence refers to the gradual deterioration of the immune system with advancing age and is true for both arms of immunity but in particular for the adaptive immune response [109]. In older people, there is a shift towards memory over naïve T cell populations [109]. Memory T cells are functionally different to naïve T cells and have a limited proliferative capacity, express fewer co-stimulatory molecules and have an altered cytokine profile [110]. B cells are also altered in the elderly and despite B cell number decreasing with age, immunoglobulin levels rise [109, 111]. These immunoglobulins are derived from B1 cells rather than B2 cells and have a low affinity for antigens [111]. B1 cells are also associated with increases in IL-6 [111]. Finally, DCs are implicated in age-related adaptive immune dysfunction as they limit the secretion of IFNs, generate an irregular immune response towards self-antigens and lose their ability to activate T cell responses [112]. As more than half of the patients admitted to the ICU are over the age of 65, immune-senescence poses a huge challenge for treating those older patients with sepsis [113].

### Sepsis heterogeneity

Sepsis heterogeneity remains one of the biggest barriers to finding an effective therapy. Transcriptome analysis of leukocytes in the peripheral blood of sepsis patients has identified 2 phenotypes in sepsis patients, assigned as sepsis response signatures (SRS)1 or SRS2 [114]. Patients with the SRS1 phenotype were immunosuppressed with indications of T cell exhaustion and low leukocyte HLA-DR expression, had low immune tolerance and also exhibited higher, early mortality than the SRS2 phenotype [114]. The study identified seven genes that were able to predict the classification of patients as SRS1 or SRS2 [114]. Other biomarkers that can indicate the immune state in sepsis patients include apoptosis markers (Bim, Bid and Bac), caspases, leukocyte markers (HLA-DR, PD-1, FOXP3, CD127) and cytokines (IL-2, IL-6, IL-12, IL-17, IL-22, IL-33, TNF-α, IFN-γ, IL-10 and TGF-β). Identifying subsets of sepsis patients most likely benefit from targeted and novel immunotherapies could be key to discovering effective therapies for this syndrome.

### Pathogen identification via host response

Pathogen identification in sepsis patients may also help predict the ensuing sepsis response and provide a better course for management with supportive care. Hyper-inflammation and/or immune suppression can occur in sepsis regardless of the source of pathogen, i.e. whether it is bacterial, fungal or viral, and regardless of the site of infection [107, 115]. Bacterial and fungal sepsis generally results in the activation of pathogen recognition receptors (PRRs) by pathogen-associated molecular patterns (PAMPs). Intracellular signalling proceeds to initiate pro-inflammatory cytokine production and additional inflammatory cell recruitment [20, 115, 116]. Interestingly, studies have shown that cytokine profiles can differ and are dependent on whether the source of infection is caused by Gram-positive or Gram-negative bacteria [117]. In relation to viral insults, the host immune response is initiated by the activation of Type I, α/β IFNs and IFN-γ, which then initiate pro-inflammatory cytokine and chemokine signalling [118, 119]. A number of viruses however can downregulate the activation of IFNs and initiate sepsis in a similar manner to non-viral pathogens [120], such as the influenza virus for example, which leads to an inflammatory state through the upregulation of cytokines [120].

## Conclusion

From the initial failed studies of strategies to suppress an assumed ‘overactive’ sepsis immune response, to findings of lymphocyte depletion and exhaustion even in earlier phase sepsis, it is now clear that in order to successfully improve sepsis outcome and improve long-term survival rates, immune homeostasis needs to be regenerated. In light of our growing understanding of the pathogenesis of sepsis, relatively new therapies that involve immunomodulating strategies (e.g. mesenchymal stem cells [121]) are showing great potential in early phase studies and may be promising contenders for treatment of this devastating syndrome if later phase trials prove successful.

## Data Availability

Not applicable

## Abbreviations

ALCs: Absolute lymphocyte counts ARDS Acute respiratory distress syndrome DCs Dendritic cells γδ gamma delta HCMV Human cytomegalovirus ICU Intensive care units IFN Interferon IL Interleukin mAbs Monoclonal antibodies NK Natural killer cells PD-L1 Programmed cell death ligand 1 Th T helper TNF Tumour necrosis factor Tregs Regulatory T cells SRS Sepsis response signatures
